# Probiotics improve intestinal ischemia–reperfusion injury: a systematic review and meta-analysis

**DOI:** 10.3389/fmed.2025.1546650

**Published:** 2025-05-22

**Authors:** Zicen Zhao, Yuxuan Wu, Yufang Leng, Liya Chang, Yu Wang, Dongbin Li, Yang Xing

**Affiliations:** ^1^The First Clinical Medical College, Lanzhou University, Lanzhou, China; ^2^Clinical Medicine Department, Fenyang College of Shanxi Medical University, Fenyang, China; ^3^The Department of Anesthesiology, The First Hospital of Lanzhou University, Lanzhou, China

**Keywords:** intestine, ischemia-reperfusion injury, probiotics, systematic review, meta-analysis

## Abstract

**Background:**

Intestinal ischemia–reperfusion injury (IRI) is a common complication of intestinal surgery and carries the risk of patient death. The treatment of intestinal IRI is an important direction of current research. This study aimed to analyze animal experiments and thus investigate the effects of probiotics administration on intestinal IRI and its mechanisms.

**Methods:**

We included a total of 12 studies from 5 databases (PubMed, Web of Science, Cochrane, Embase, and Scopus), incorporating outcome metrics including Chiu’s score, levels of malondialdehyde (MDA), myeloperoxidase (MPO) tumor necrosis factor-alpha (TNF-*α*), IL-6, IL-1β, occludin, zonula occludens-1 (ZO-1), FITC-dextran and intestinal bacteria (Lactobacillus, Bacteroides and Bifidobacteria). Statistical analysis was performed using Review Manager 5.3.

**Results:**

We found that probiotic-added animals had less intestinal damage after IRI compared to controls, as evidenced by a more intact intestinal barrier [occludin (2.83, 95% CI: 1.46 to 4.20, *p* < 0.0001), ZO-1 (3.30, 95% CI: 1.58 to 5.01, *p* = 0.0002) and FITC-dextran (−3.83, 95% CI: −5.83 to −2.29, *p* < 0.0001)], lower Chiu score (−1.83, 95% CI: −2.47 to −1.20, *p* < 0.0001), fewer inflammatory factors [IL-6 (−2.19, 95% CI: −3.98 to −0.39, *p* = 0.02), TNF-*α* (−2.24, 95% CI: −4.15 to −0.33, *p* = 0.02)], and lower levels of oxidative stress [MDA (−2.46, 95% CI: −4.62 to −0.30, *p* = 0.03), MPO (−0.97, 95% CI: −1.77 to −0.17; *p* = 0.02)]. In addition, probiotic supplementation increased the relative abundance of Lactobacillus (0.90, 95% CI: 0.33 to 1.48, *p* = 0.002) and Bacteroides (0.81, 95% CI: 0.14 to 1.49, *p* = 0.02), thus maintaining the stability of the gut microbiota.

**Conclusion:**

In conclusion, the mechanisms by which probiotic therapy attenuates intestinal IRI may be related to decreased levels of inflammation and oxidative stress, increased probiotic abundance, and increased expression of tight junction (TJ) proteins.

**Systematic Review Registration:**

https://www.crd.york.ac.uk/PROSPERO/view/CRD42024577459.

## 1 Introduction

Intestinal IRI is a severe clinical condition that frequently arises as a complication of intestinal surgeries and critical illnesses, such as traumatic shock, severe burns, and strangulated bowel obstruction (1). The pathophysiology of intestinal IRI involves two distinct phases: an ischemic phase, during which blood flow to the intestines is restricted, and a reperfusion phase, during which the restoration of blood flow paradoxically exacerbates tissue damage. This process results in intestinal epithelial cell injury, oxidative stress, disruption of the intestinal barrier, interstitial edema, and the release of inflammatory mediators (2). These events can trigger systemic inflammatory response syndrome (SIRS), which may progress to multi-organ dysfunction and even death (3). Despite advances in surgical techniques and critical care management, intestinal IRI remains associated with high morbidity and mortality rates, underscoring the urgent need for effective therapeutic interventions.

Recent studies have highlighted the pivotal role of the gut microbiota in the pathogenesis of intestinal IRI (4). Ischemia–reperfusion disrupts the balance of the intestinal microbiota, leading to dysbiosis characterized by the overgrowth of pathogenic bacteria, such as *Escherichia coli*, and a reduction in beneficial bacteria, such as Lactobacillus. This microbial imbalance exacerbates intestinal inflammation by activating the immune system and promoting a strong pro-inflammatory response (1). Probiotics, as modulators of the gut microbiome, have emerged as a promising therapeutic strategy to counteract these effects. Probiotics are commensal microorganisms that naturally reside in the gastrointestinal tract, with major genera including Lactobacillus, Bifidobacterium, Enterococcus, and *Escherichia coli* (5, 6). These beneficial microbes play a critical role in maintaining intestinal homeostasis by inhibiting the colonization of pathogenic bacteria, restoring microbial balance, enhancing intestinal barrier function, and modulating immune responses (7). In recent years, probiotics have gained increasing attention for their ability to modulate the immune system and maintain intestinal integrity, leading to their widespread use in the prevention and treatment of various diseases (8). Notably, probiotics have demonstrated significant potential in mitigating the effects of intestinal IRI. For instance, *Bifidobacterium longum* has been shown to attenuate IRI-induced inflammation and oxidative stress by inhibiting the NF-κB inflammatory pathway and activating the Keap1/Nrf2 signaling pathway, thereby reducing intestinal tissue damage in rat models (9). These findings highlight the therapeutic potential of probiotics in managing IRI and its associated complications.

Although some studies have investigated the role of probiotics in intestinal IRI, the current body of evidence remains limited, and the precise mechanisms underlying their protective effects are not yet fully understood. Therefore, this meta-analysis aims to systematically evaluate the effects of probiotic therapies in animal models of intestinal IRI. By synthesizing the available evidence, this study seeks to establish a foundation for future clinical trials and to inform the development of novel therapeutic strategies targeting the gut microbiome for the effective management of intestinal IRI.

## 2 Methods

This study has been registered with the Prospective Registry for Systematic Reviews platform (PROSPERO; registration ID: CRD42024577459), and all relevant information and modifications have been documented.

### 2.1 Search strategy

We conducted a comprehensive literature search in the following databases: PubMed, Web of Science, Cochrane Library, Embase, and Scopus. The search utilized the terms “ischemia–reperfusion injury, ““intestines, “and “probiotics” to retrieve all relevant literature (as detailed in Supplementary Table 1). The search was performed independently by two authors, Zicen Zhao and Yuxuan Wu.

### 2.2 Inclusion and exclusion criteria

Inclusion criteria: (1) Studies that utilized models of intestinal ischemia–reperfusion involving superior mesenteric artery occlusion (regardless of species or gender); (2) Research that was designed to examine changes in factors related to probiotics. Exclusion criteria: (1) Studies that involved animals with pre-existing comorbidities; (2) *In vitro* studies or computer simulations; (3) Intestinal ischemia–reperfusion models that did not employ superior mesenteric artery occlusion; (4) Studies that lacked accessible data or from which data could not be extracted; (5) Duplicate publications.

### 2.3 Data collection

Data were independently extracted from each relevant article by two authors, Zicen Zhao and Yuxuan Wu, using a standardized data extraction form that was developed for this purpose. The authors initially attempted to extract numerical data from the text, tables, or figures. If data were not reported in these formats, WebPlotDigitizer was employed to extract information from graphical representations. The extracted data included the following:

*Study information*: First author, year of publication, language, and country.

*Animal characteristics*: Species, gender, age, and weight.

*Model details*: Duration of ischemia–reperfusion (including clamping and unclamping times of the superior mesenteric artery), anesthetic agents used, probiotic strains administered, dosages, and intervention methods.

*Outcome measures*: Chiu’s score, intestinal levels of MDA, MPO, occludin, ZO-1, FITC-dextran and intestinal microbiota composition (Lactobacillus, Bacteroides, and Bifidobacteria), as well as serum levels of IL-1β, IL-6 and TNF-*α*.

### 2.4 Data analysis

#### 2.4.1 Risk of bias assessment

Two authors, Liya Chang and Dongbin Li, independently evaluated the included studies using the Systematic Review Center for Laboratory Animal Experiments (SYRCLE) risk of bias tool (10). Any disagreements between the authors were resolved through discussion or with input from a third author, Yang Xing.

#### 2.4.2 Assessment of heterogeneity

Heterogeneity was assessed using the I^2^ statistic. An I^2^ value of ≤ 50% indicated small heterogeneity, prompting the use of a fixed-effects model. Conversely, an I^2^ value of > 50% suggested significant heterogeneity, warranting the application of a random-effects model. An I^2^ value of ≥ 75% was considered substantial heterogeneity (11).

#### 2.4.3 Data analysis

Data were synthesized using Review Manager (RevMan) version 5.4 software. The standardized mean difference (SMD) with a 95% confidence interval (CI) was employed to analyze continuous data (11). In instances where I^2^ > 50%, potential sources of heterogeneity were explored through subgroup analyses and sensitivity analyses within the meta-analysis framework. If I^2^ ≥ 5% and the cause of heterogeneity remained undetermined, a descriptive analysis was conducted. Additionally, funnel plots were generated when more than 10 studies were included to assess publication bias.

## 3 Results

### 3.1 Study selection

**Figure 1:**
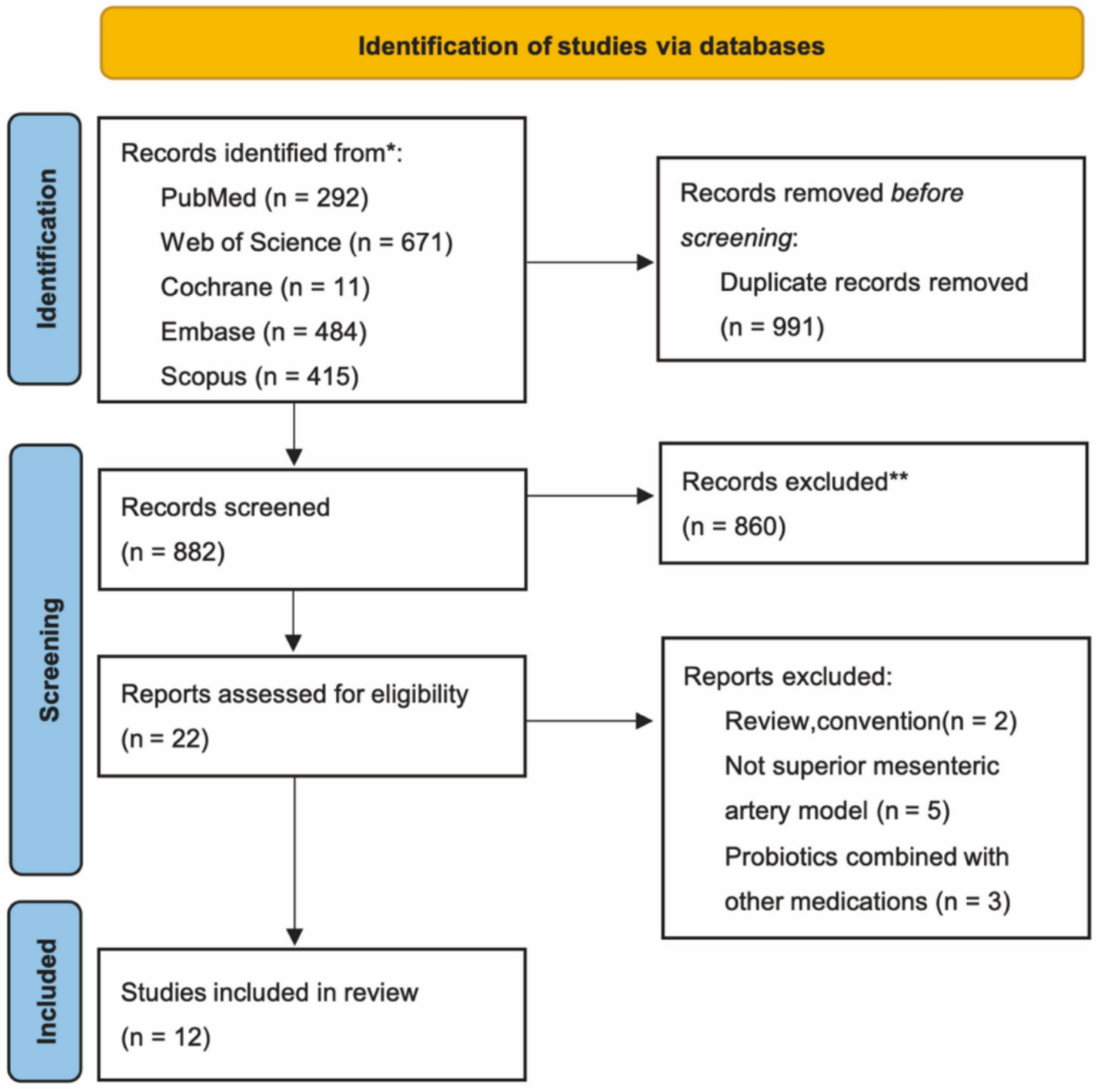
Article screening flowchart.

A total of 1873 articles were retrieved from the five databases. Following the removal of 991 duplicates, 860 articles were excluded after reviewing titles and abstracts. The remaining 22 articles were assessed in full independently by two authors. These articles were evaluated based on the predetermined inclusion and exclusion criteria, resulting in the inclusion of 12 studies (9, 12–22). The retrieval process is illustrated in Figure 1.

### 3.2 Basic characteristics of the included studies

The final analysis comprised 12 studies involving a total of 186 animals. We summarized the key characteristics of the included studies, which are detailed as follows: (1) first author; (2) year of publication; (3) language; (4) species and gender of the animals; (5) age and weight of the animals; (6) duration of ischemia/reperfusion; (7) anesthetic agents used (Table 1).

**Table 1:** Characteristics of included studies.

Name	Country	Language	Species /Gender	Age /Weight	I/R duration	Anesthetic	Probiotic strains/Dosage	Intervention method
Jakesevic 2011 (12)	Sweden	English	BALBcJ /Male	U /20 g	30 min/4 h	7.5 mg Ketamine (50 mg/mL) and 2.5 mg Xylazine (20 mg/mL)/100 g	*L. plantarum* HEAL19/1·10^8^ cfu per day	Feed
Wang 2013 (13)	China	English	BALBc /Male	6 ~ 8w /20 ~ 25 g	60 min/3 h	Ketamine (120 mg/kg, intraperitoneally)	Bifidobacteria/1·10^9^ cells in 0.2 mL for 2w	Oral ingestion
Duranti 2018 (14)	Italy	English	Swiss /Female	U /18 ~ 25 g	45 min/5 h	Pentobarbital (70 mg/kg i.p.)	*B. bifidum* PRL2010/10^9^ cells per day for 5d	U
Hu 2022 (15)	China	English	C57BL6J /Male	6 ~ 8w /20 ~ 25 g	60 min/2 h	4% Isoflurane	*Lactobacillus murinus*/7d with 200 μL 6.8 × 10^8^ CFU	Gavage
Wang 2011 (16)	China	English	SD /Female	6 mth /180 ~ 220 g	30 min/4 h	Ketamine (100 mg/kg, i.p.)	*Lactobacillus plantarum* L2/14d with 2*10^10^ CFU	Gavage
Håkansson 2006 (17)	Sweden	English	BALBcJ /Male	U /20 g	30 min/4 h	7.5 mg Ketamine (50 mg/mL) and 2.5 mg Xylazine (20 mg/mL)/100 g	*L. plantarum* DSM 9843/1 mL,10^9^ CFU	U
Salim 2013 (18)	Canada	English	129SvEv /Male	U	60 min/2 h	ketamine (120 mg/kg) and xylazine (16 mg/kg)	VSL#3 probiotics/3 mg/mL	Gavage
Gao 2024 (19)	China	English	C57BL6J /Male	6 ~ 8w /U	30 min/U	Isoflurane	*Akkermansia muciniphila* 200 μL 1*10^9^ CFUs/mL	Oral ingestion
Takayama 2022 (20)	Japan	English	ICR/ Male	5w/U	30 min /24 h	ketamine (100 mg/kg) and xylazine (10 mg/kg)	*Lactobacillus bulgaricus* and Streptococcus thermophiles	Feed
Tang 2023 (21)	China	English	SD /Male	8w/U	45 min/2 h	U	Lactiplantibacillus plantarum GL001 1*10^9^ CFU/mL per day	Oral ingestion
Tang 2024 (9)	China	English	SD /Male	8w/U	45 min/2 h	U	*Bifidobacterium longum* GL001 1*10^9^ CFU/mL per day	Oral ingestion
Chen 2024 (22)	China	English	C57BL6J /Male	8–10w/U	30 min/6 h	2.5% 2,2,2-Tribromoethanol 100–150 μL	*Saccharomyces cerevisiae* (ATCC204991) 108 CFU	Gavage

SD, Sprague–Dawley; ICR, Institute of Cancer Research; U, unclear; min, minute(s); h, hour.

### 3.3 Quality assessment of research

**Figure 2:**
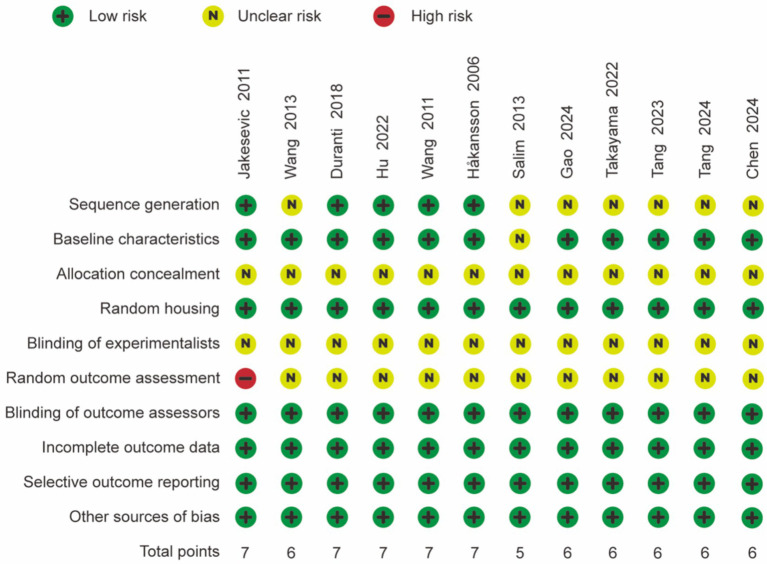
Overall quality of included studies assessed with SYRCLE risk of bias assessment tool.

The quality of the 12 studies was assessed independently using the 10-item system outlined in the SYRCLE risk of bias tool, and the results are presented in Figure 2. The majority of the studies received scores of 6 or above, indicating satisfactory study quality. All animals were randomized, and complete outcome data were reported in each study, with no indication of selective reporting. However, half of the studies were classified as having unclear randomization. None of the studies specified the methods of blinding or concealment of allocation order implemented for trial caregivers and researchers. Overall, the included studies demonstrated moderate quality.

### 3.4 Probiotic supplementation attenuated ischemia–reperfusion-induced intestinal injury

**Figure 3:**
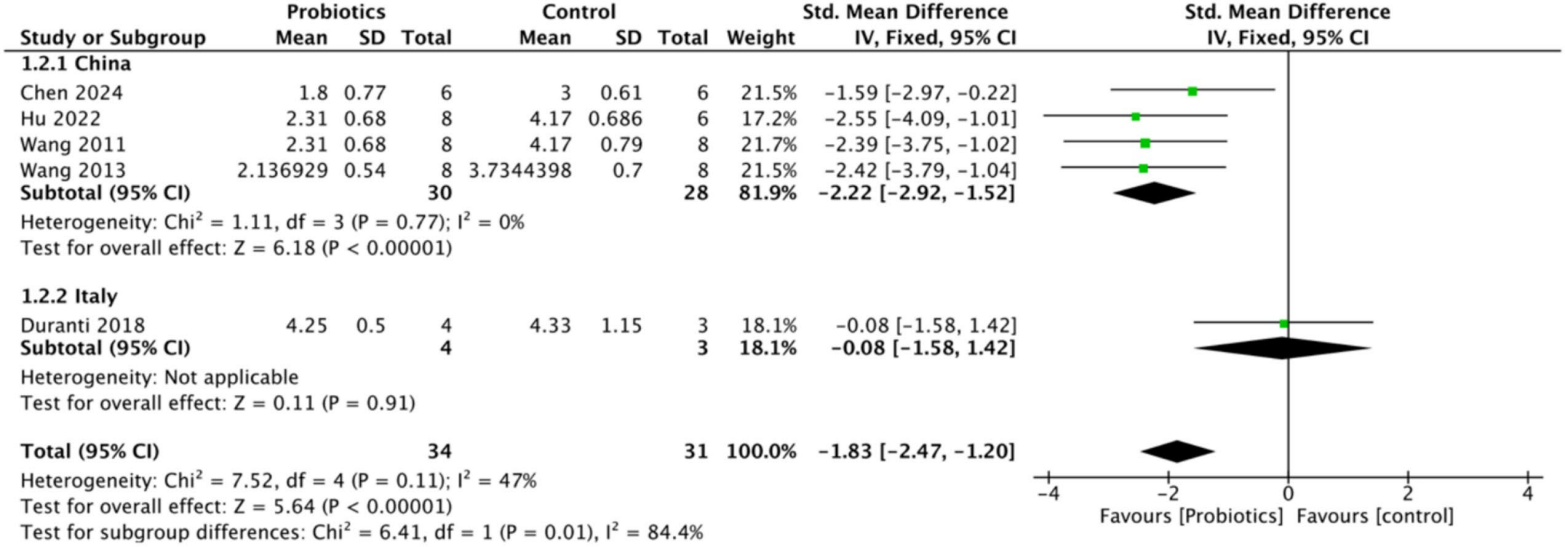
Forest plot of meta-analysis of all studies testing Chiu’s score.

To assess the impact of probiotics on intestinal ischemia–reperfusion injury, Chiu’s score was selected as the primary outcome measure. Five studies evaluated Chiu’s score, revealing a significant reduction in the probiotic group compared to the control group, with a mean difference of (−1.83, 95% CI: −2.47 to −1.20, *p* < 0.0001), suggesting that probiotics attenuated intestinal tissue damage. Notably, heterogeneity (I^2^ = 0) decreased when Chiu’s score was analyzed by the country of study, indicating that geographic variability may have contributed to the heterogeneity observed in Chiu’s score (Figure 3).

### 3.5 Effectiveness of secondary indicators

We examined intestinal levels of MDA, MPO, occludin, ZO-1 and FITC-dextran, as well as the composition of the intestinal microbiota, including Lactobacillus, Bacteroides, and Bifidobacterium. In addition, we assessed serum levels of IL-1β, IL-6, and TNF-*α*. These indicators were used as secondary outcome measures.

#### 3.5.1 Probiotic supplementation attenuated ischemia–reperfusion-induced oxidative stress

**Figure 4:**
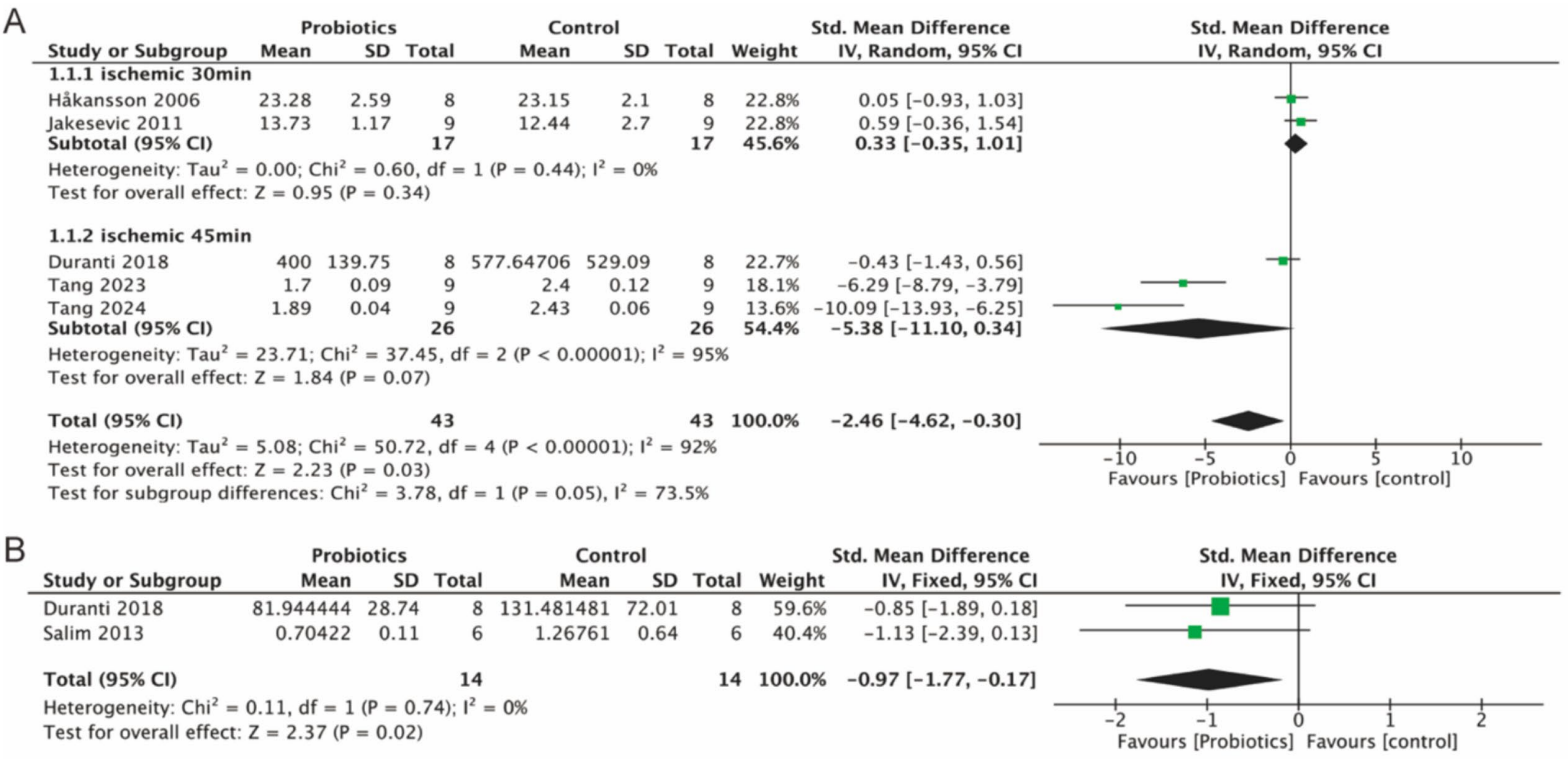
Forest plot of meta-analysis of all studies detecting MDA and MPO. **(A)** Forest plot of MDA levels in intestinal tissue. **(B)** Forest plot of MPO levels in intestinal tissue.

Five studies assessed MDA as a marker of oxidative stress. MDA served as a measure of oxidative stress, and our analysis of all literature measuring MDA indicated that MDA levels decreased in the probiotic group compared to the control group (−2.46, 95% CI: −4.62 to −0.30, *p* = 0.03) (Figure 4A). Additionally, elevated levels of MPO were associated with increased oxidative stress. Two studies analyzed MPO levels, and the probiotic group demonstrated a reduction in MPO expression (−0.97, 95% CI: −1.77 to −0.17; *p* = 0.02) compared to controls (Figure 4B).

#### 3.5.2 Probiotic supplementation attenuated ischemia–reperfusion-induced intestinal barrier disruption

**Figure 5:**
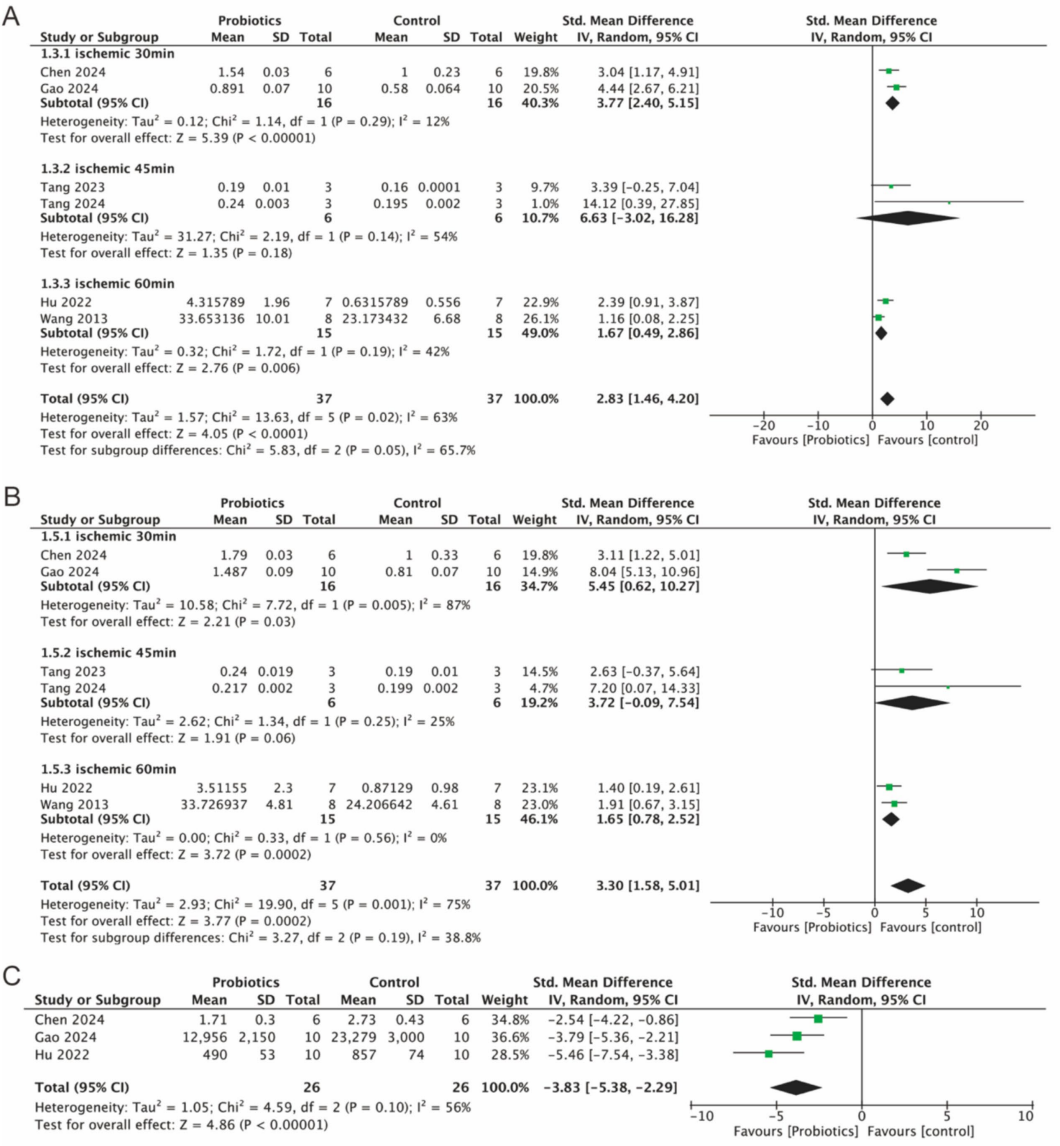
Forest plot of meta-analysis of all studies detecting occludin, ZO-1 and FITC-dextran. **(A)** Forest plot of occludin. **(B)** Forest plot of ZO-1. **(C)** Forest plot of FITC-dextran.

Six studies evaluated the expression of tight junction (TJ) proteins (occludin and ZO-1), three studies examined the levels of FITC-dextran, an indicator of intestinal permeability. Integrated analyses demonstrated a significant increase in the expression levels of occludin (2.83, 95% CI: 1.46 to 4.20, *p* < 0.0001) and ZO-1 (3.30, 95% CI: 1.58 to 5.01, *p* = 0.0002) in the probiotic group compared to controls (Figures 5A,B). Figure 5C showed that the level of FITC-dextran (−3.83, 95% CI: −5.83 to −2.29, *p* < 0.0001) was significantly lower in the probiotic group compared to the control group, which suggested that intestinal permeability was reduced and the intestinal barrier was protected by probiotic supplementation. In addition, subgroup analyses revealed a reduction in the heterogeneity of the occludin parameter, while the heterogeneity of the ZO-1 parameter decreased in the 45-min and 60-min ischemia groups. This suggests that different ischemic durations may contribute to the heterogeneity observed in the detection of TJ proteins.

#### 3.5.3 Probiotic supplementation increased the relative abundance of intestinal Lactobacillus and Bacteroides

**Figure 6:**
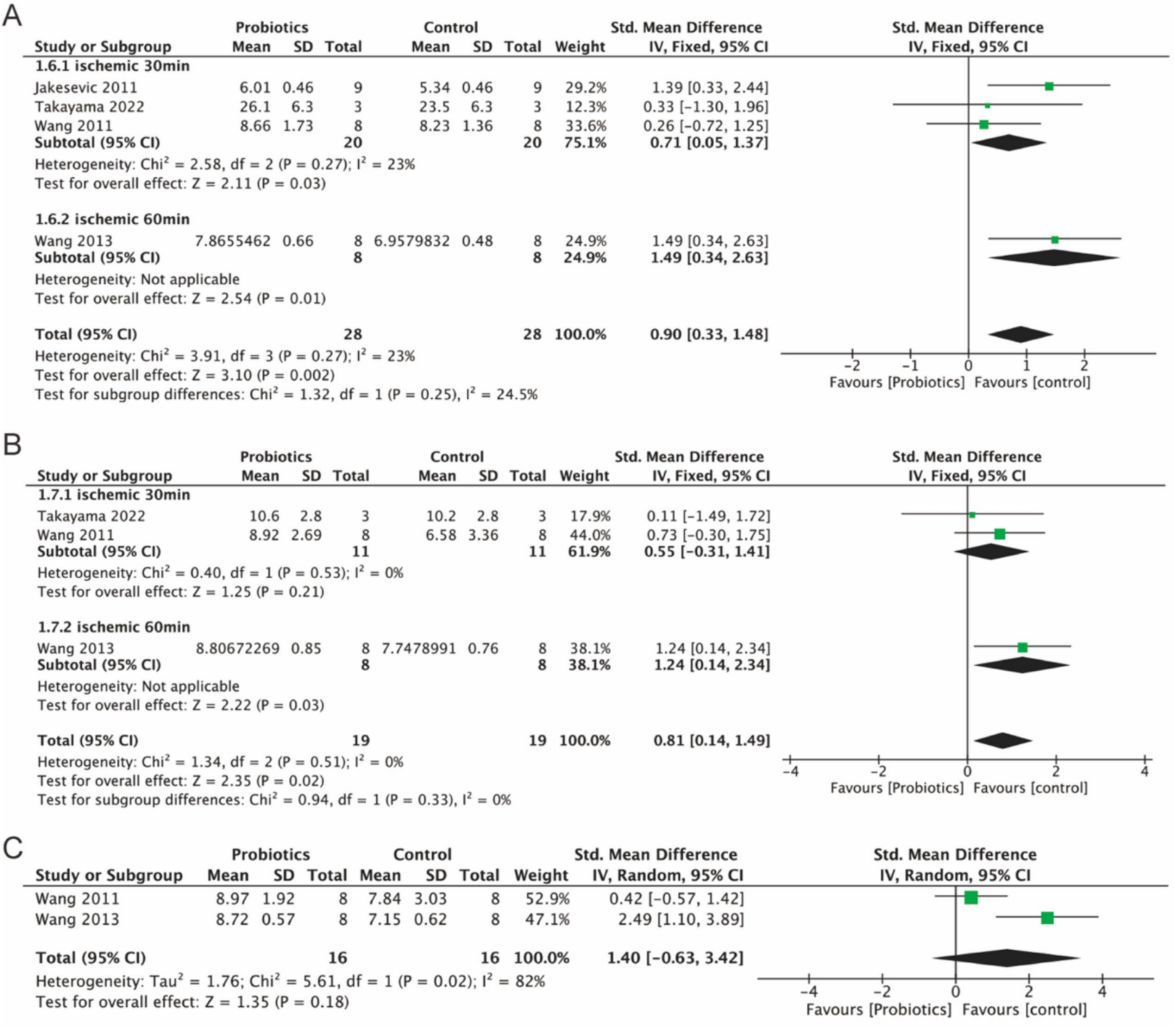
Forest plot of meta-analysis of all studies detecting Lactobacillus, Bacteroides and Bifidobacteria. **(A)** Forest plot of Lactobacillus. **(B)** Forest plot of Bacteroides. **(C)** Forest plot of Bifidobacteria.

Four studies investigated the relative abundance of Lactobacillus, three studies assessed the levels of Bacteroides, and two studies examined the levels of Bifidobacteria. The findings indicated a slight increase in the relative abundance of Lactobacillus (0.90, 95% CI: 0.33 to 1.48, *p* = 0.002) and Bacteroides (0.81, 95% CI: 0.14 to 1.49, *p* = 0.02) in the probiotic group. Although Bifidobacteria exhibited an increase in relative abundance (1.40, *p* = 0.18), this result was not statistically significant (Figures 6A–C).

#### 3.5.4 Probiotic supplementation reduced serum inflammatory factor levels

**Figure 7:**
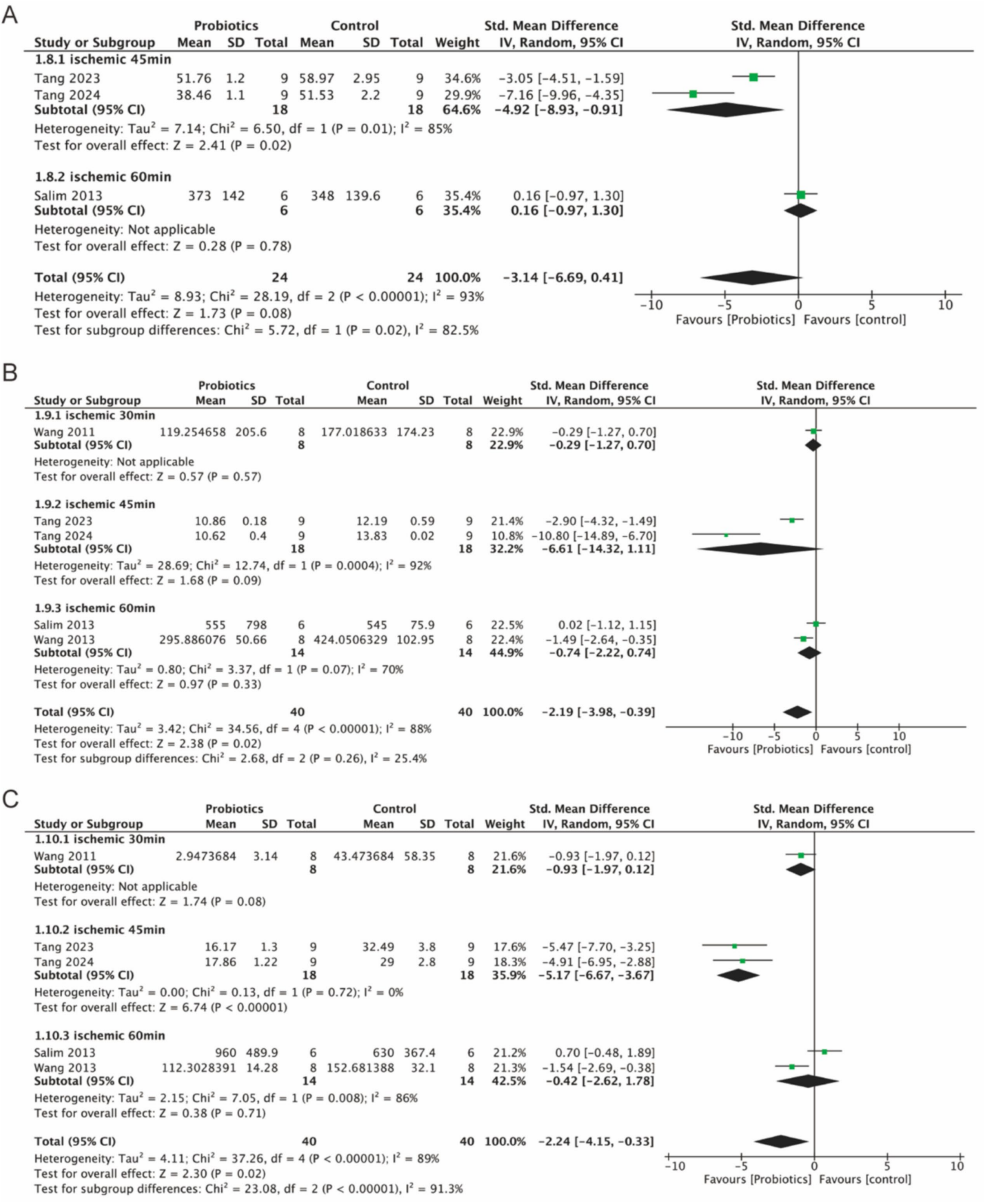
Forest plot of meta-analysis of all studies detecting IL-1β, IL-6 and TNF-*α*. **(A)** Forest plot of IL-1β. **(B)** Forest plot of IL-6. **(C)** Forest plot of TNF-α.

Three studies evaluated serum levels of IL-1β, while five studies assessed serum levels of TNF-*α* and IL-6. The probiotic group demonstrated a significant reduction in serum IL-6 (−2.19, 95% CI: −3.98 to −0.39, *p* = 0.02) and TNF-α (−2.24, 95% CI: −4.15 to −0.33, *p* = 0.02) compared to the control group (Figures 7B,C). Although IL-1β levels were reduced in the probiotic group, the decrease was not statistically significant (*p* = 0.08) (Figure 7A). However, subgroup analysis based on ischemia duration revealed a statistically significant reduction in IL-1β levels in the 45-min ischemia group (−4.92, 95% CI: −8.93 to −0.91, *p* = 0.02).

## 4 Discussion

This review provides a comprehensive summary of all preclinical studies investigating the efficacy of probiotics for the treatment of intestinal IRI. While animal experiments have inherent limitations, the findings still provide valuable guidance for the design of clinical trials. Our review included 12 different studies that examined the beneficial effects of probiotics on intestinal IRI. The results showed that animals with intestinal IRI showed an increase in the relative abundance of intestinal bacteria, especially Lactobacillus and Lactobacillus, after probiotic supplementation; the integrity of the intestinal barrier was also protected to some extent, with a significant reduction in intestinal mucosal damage and a lessened inflammatory response. The following are possible mechanisms by which probiotics ameliorate intestinal damage:

### 4.1 Anti-oxidative stress

Oxidative stress, characterized by an imbalance between pro-oxidants and antioxidants, plays a pivotal role in the pathophysiology of intestinal IRI. It enhances neutrophil infiltration and leads to increased secretion of pro-inflammatory proteins. During ischemic episodes, mitochondria generate substantial quantities of reactive oxygen species (ROS), including superoxide anions (O2·−), hydrogen peroxide (H2O2), and hydroxyl radicals (·OH) (23, 24). Upon restoration of blood flow to the intestinal tissue, a significant influx of oxygen activates oxidative stress through ROS, thereby initiating a cascade of signaling pathways and inflammatory responses (25).

Lipids represent a major class of molecules affected by oxidative stress, undergoing peroxidation and yielding various secondary products, with MDA serving as a common biomarker for oxidative stress and cellular membrane damage (24). Our findings demonstrated that probiotics lowered MDA levels and alleviated oxidative stress damage in intestinal tissues subjected to IRI (9, 14, 21). Moreover, probiotic supplementation was shown to reduce levels of MPO (14, 18) and ROS (9, 21). Elevated MPO activity promoted ROS production, thereby exacerbating lipid peroxidation and elevating MDA levels (26). The correlations observed in these parameters underscore a significant relationship between probiotic therapy and the management of oxidative stress in the context of intestinal IRI.

### 4.2 Protection of the intestinal barrier

The intestinal epithelial cells are integral to maintaining a barrier that facilitates nutrient absorption while simultaneously protecting the body from microbial invasion (27). The functionality of this epithelial barrier is critically dependent on TJ proteins, which are essential for the integrity of the mechanical barrier. Intestinal IRI can compromise this barrier through various mechanisms, including the action of pro-inflammatory cytokines and the augmented production of ROS, resulting in increased intestinal permeability and a reduction in TJ proteins such as ZO-1 and occludin (28, 29).

Occludin and ZO-1 are vital components of TJ proteins that regulate the proliferation and survival of epithelial cells. Notably, the overexpression of occludin has been shown to enhance epithelial barrier function (30). ZO-1, a member of the membrane-associated guanylate kinase homolog family, plays a crucial role in connecting TJ membrane proteins to the cytoskeleton, in collaboration with ZO-2 and ZO-3 (31). Probiotics have been demonstrated to have beneficial effects on the integrity of the intestinal barrier, for instance, *Lactobacillus plantarum* HNU082 alleviates ulcerative colitis by modulating gut microbiota and its metabolites, leading to increased expression of ZO-1 and occludin (32). This protective effect is facilitated by the U5 domain of probiotics, which is essential for maintaining the integrity of apical membranes and microvilli (33). In our compilation of studies examining the effects of probiotics on animals with intestinal IRI, results showed that probiotic supplementation led to a significant increase in the levels of TJ proteins ZO-1 and occludin (9, 13, 15, 19, 21), thereby enhancing the integrity of the intestinal barrier and confirming the beneficial impacts of probiotics.

In addition, FITC-dextran, diamine oxidase (DAO), and D-lactic (D-LAC) serve as important biomarkers for assessing intestinal barrier function. A compromised intestinal barrier permits the translocation of intestinal bacteria and undigested food components into the bloodstream, leading to elevated serum levels of DAO (34). Similarly, the presence of elevated D-lactic acid, a byproduct of bacterial fermentation, and increased levels of FITC-dextran, a macromolecular glycan, indicate impaired intestinal barrier function (35, 36). Therefore, the monitoring of these biomarkers is crucial for evaluating intestinal barrier integrity. Our results indicate that probiotic treatment significantly reduced the levels of FITC-dextran (15, 19, 22), serum DAO, and D-LAC (9, 21), thereby protecting the intestinal mucosal barrier from IRI-induced functional impairment.

### 4.3 Regulation of intestinal flora abundance

The intestinal microbiota comprises a diverse array of microorganisms that play a crucial role in gut health and are closely associated with intestinal IRI. Major microbial groups within the intestinal flora include Lactobacillus, Bifidobacterium, Bacteroides, and *Escherichia coli*, among others (37). Intestinal IRI disrupts the microenvironment, resulting in notable alterations in microbial composition, characterized by an increase in pathogenic organisms such as *E. coli* and *Clostridium difficile*, alongside a reduction in beneficial flora like Lactobacillus (1).

Maintaining the stability of the intestinal microbiota is essential for preserving gut homeostasis. Our findings indicated that probiotics significantly enhanced the abundance of beneficial genera, notably Lactobacillus, Bifidobacterium, and Bacteroides (12, 13, 16, 20). Specifically, probiotics were shown to increase *Lactobacillus murinus* while concurrently decreasing levels of Prevotella (15, 20). These regulatory effects contribute to the maintenance of a balanced intestinal microenvironment, reducing bacterial translocation and mitigating epithelial cell damage in intestinal tissues subjected to ischemia–reperfusion.

### 4.4 Anti-inflammatory

The inflammatory response serves as a critical mechanism in the pathophysiology of intestinal IRI. During episodes of IRI, there is a marked infiltration of leukocytes and neutrophils, which produce a range of chemokines and inflammatory mediators that compromise intestinal barrier function (38). This process activates pivotal cellular signaling pathways, including the nuclear factor kappa B (NF-κB) and mitogen-activated protein kinase (MAPK) pathways. In the NF-κB signaling pathway, the IκB kinase complex becomes activated, leading to the phosphorylation and degradation of the IκB protein. This action allows NF-κB transcription factors to translocate to the nucleus, where they form active transcription complexes (39). Conversely, the MAPK pathway is triggered via the Raf–MEK–ERK cascade, facilitating the phosphorylation of various MAPKs (e.g., ERK, JNK, p38), which then enter the nucleus to activate transcription factors (40, 41). Activation of these pathways ultimately results in increased mRNA expression of pro-inflammatory cytokines, such as tumor necrosis factor-alpha (TNF-*α*), interleukin-6 (IL-6), and interleukin-1 beta (IL-1β). These mediators then enter the systemic circulation, fostering a systemic inflammatory response (39, 42). Our studies demonstrated that probiotics mitigated inflammatory damage induced by ischemia–reperfusion by reducing the expression of pro-inflammatory cytokines, including TNF-α, IL-6, and IL-1β (9, 13, 16, 21). Notably, a study by Salim et al. reported a slight increase in TNF-α, IL-6, and IL-1β levels post-probiotic supplementation compared to the control group; however, this increase was not statistically significant (18). This discrepancy may be attributed to the use of a mixed probiotic formulation in Salim’s study rather than a focused single strain approach.

In summary, probiotics can exert protective effects on intestinal IRI animal models through multiple mechanisms. However, due to the potential differences between animal models and humans in terms of immune system response, gut microbiota composition, and intestinal barrier function, there are still many challenges in applying probiotics to the clinical improvement of injuries caused by intestinal ischemia in patients, and more clinical trials are needed to verify its feasibility.

## 5 Advantages and limitations of this review

### 5.1 Strengths

This review has several notable strengths. First, it represents the first meta-analysis of preclinical studies investigating the effects of probiotics on intestinal ischemia–reperfusion injury. Second, we comprehensively evaluated the role of probiotics in various aspects, including oxidative stress, intestinal barrier integrity, gut microbiota composition, and inflammatory factors. This multifaceted approach provides a valuable reference for future preclinical studies and clinical treatments related to intestinal IRI.

### 5.2 Limitations

Despite its strengths, this review has certain limitations. First, the studies included varied in terms of animal models, duration of modeling, and types of probiotic strains used, which may have influenced the results to some extent. Second, the overall number of animals included in the studies reviewed is relatively small, which may impact outcomes. Last, the limited number of studies included in this review precluded the possibility of assessing publication bias, hindering further validation of our findings.

## 6 Conclusion and perspectives

In conclusion, this review evaluated the effects of probiotic supplementation on intestinal IRI in animal studies. The results indicate that probiotic therapy reduces inflammation and oxidative stress, enhances the expression of TJ proteins—particularly occludin and ZO-1—and increases the relative abundance of beneficial intestinal bacteria, thereby mitigating the impact of intestinal IRI. Moreover, these findings offer valuable insights for the design of clinical trials, particularly concerning the use of probiotics from Lactobacillus and Bacteroides to elective intestinal surgery to prevent ischemia–reperfusion injury in the intestines during the surgical procedure.

In addition to these findings, the studies included in this review also examined the effects of probiotics on various other factors. Notably, one study found that probiotic supplementation elevated the levels of several short-chain fatty acids (SCFAs), including acetate, propionate, butyrate, and valerate, in mice compared to controls. This suggests that probiotic therapy may significantly influence SCFA production, indicating that the underlying mechanisms merit further investigation. These insights could offer new perspectives for clinical trials and therapeutic approaches targeting intestinal IRI.

## Data Availability

Publicly available datasets were analyzed in this study. This data can be found at: the authors will supply the relevant data in response to reasonable requests.
