# Supplementary material for: Probiotics improve intestinal ischemia–reperfusion injury: a systematic review and meta-analysis
Source: Front Med (Lausanne). 2025 May 22;12:1546650. doi: 10.3389/fmed.2025.1546650 (PMC12142053; doi:10.3389/fmed.2025.1546650)
Supplement: Supplementary file 1 [file Table_1.DOCX]

***Supplementary Material***

Supplementary Table 1. Probiotics act on intestinal ischemia-reperfusion injury in a searchable format

|  |  | Search | Results |
| --- | --- | --- | --- |
| PubMed |  | ((((((((((((((Reperfusion Injury) OR (Reperfusion Injuries)) OR (Injury, Ischemia-Reperfusion)) OR (Injury, Ischemia Reperfusion)) OR (Ischemia-Reperfusion Injuries)) OR (Injury, Reperfusion)) OR (Ischemia-Reperfusion Injury)) OR (Ischemia Reperfusion Injury)) OR (Reperfusion Damage)) OR (Damage, Reperfusion)) OR (Reperfusion Damages)) OR (Ischemia Reperfusion)) OR (Ischemia-Reperfusion)) AND ((((Intestines) OR (Intestine)) OR (Intestinal)) OR (gut))) AND ((((((((((((((((((((((((((((probiotics) OR (probiotic)) OR (prebiotics)) OR (synbiotics)) OR (symbiotic)) OR (Lactobacillus)) OR (Lactobacillaceae)) OR (Lactococcus)) OR (Bacillus)) OR (Clostridium)) OR (Saccharomyces)) OR (Streptococcus)) OR (Bifidobacterium)) OR (Enterococcus)) OR (Bioflor)) OR (Akkermansia)) OR (Lactiplantibacillus)) OR (Pediococcus)) OR (Lacticaseibacillus casei)) OR (Streptococcus)) OR (Leuconostoc)) OR (Clostridium butyricum)) OR (Microbiotas)) OR (Microbiota)) OR (Microbiome)) OR (Microbiomes)) OR (Mycobiomes)) OR (Mycobiome)) | 292 |
| WOS | #1 | Reperfusion Injury (Topic) or Reperfusion Injuries (Topic) or Injury, Ischemia-Reperfusion (Topic) or Injury, Ischemia Reperfusion (Topic) or Ischemia-Reperfusion Injuries (Topic) or Injury, Reperfusion (Topic) or Ischemia-Reperfusion Injury (Topic) or Ischemia Reperfusion Injury (Topic) or Reperfusion Damage (Topic) or Damage, Reperfusion (Topic) or Reperfusion Damages (Topic) or Ischemia Reperfusion (Topic) or Ischemia-Reperfusion (Topic) and Preprint Citation Index (Exclude – Database) | 204457 |
|  | #2 | Intestines (Topic) or Intestine (Topic) or Intestinal (Topic) or gut (Topic) and Preprint Citation Index (Exclude – Database) | 1273377 |
|  | #3 | probiotics (Topic) or probiotic (Topic) or prebiotics (Topic) or synbiotics (Topic) or symbiotic (Topic) or Lactobacillus (Topic) or Lactobacillaceae (Topic) or Lactococcus (Topic) or Bacillus (Topic) or Clostridium (Topic) or Saccharomyces (Topic) or Streptococcus (Topic) or Bifidobacterium (Topic) or Enterococcus (Topic) or Bioflor (Topic) or Akkermansia (Topic) or Lactiplantibacillus (Topic) or Pediococcus (Topic) or Lacticaseibacillus casei (Topic) or Streptococcus (Topic) or Leuconostoc (Topic) or Clostridium butyricum (Topic) or Microbiotas (Topic) or Microbiota (Topic) or Microbiome (Topic) or Microbiomes (Topic) or Mycobiomes (Topic) or Mycobiome (Topic) and Preprint Citation Index (Exclude – Database) | 1691204 |
|  | #4 | #1 AND #2 AND #3 | 671 |
| Cochrane | #1 | (Reperfusion Injury):ti,ab,kw OR (Reperfusion Injuries):ti,ab,kw OR (Injury, Ischemia-Reperfusion):ti,ab,kw OR (Injury, Ischemia Reperfusion):ti,ab,kw OR (Ischemia-Reperfusion Injuries):ti,ab,kw | 3792 |
|  | #2 | (Injury, Reperfusion):ti,ab,kw OR (Ischemia-Reperfusion Injury):ti,ab,kw OR (Ischemia Reperfusion Injury):ti,ab,kw OR (Reperfusion Damage):ti,ab,kw OR (Damage, Reperfusion):ti,ab,kw | 4041 |
|  | #3 | (Reperfusion Damages):ti,ab,kw OR (Ischemia Reperfusion):ti,ab,kw OR (Ischemia-Reperfusion):ti,ab,kw | 4411 |
|  | #4 | #1 OR #2 OR #3 | 5320 |
|  | #5 | (Intestines):ti,ab,kw OR (Intestine):ti,ab,kw OR (Intestinal):ti,ab,kw OR (gut):ti,ab,kw | 41596 |
|  | #6 | (probiotics):ti,ab,kw OR (probiotic):ti,ab,kw OR (prebiotics):ti,ab,kw OR (synbiotics):ti,ab,kw OR (symbiotic):ti,ab,kw | 13120 |
|  | #7 | (Lactobacillus):ti,ab,kw OR (Lactobacillaceae):ti,ab,kw OR (Lactococcus):ti,ab,kw OR (Bacillus):ti,ab,kw OR (Clostridium):ti,ab,kw | 11678 |
|  | #8 | (Saccharomyces):ti,ab,kw OR (Streptococcus):ti,ab,kw OR (Bifidobacterium):ti,ab,kw OR (Enterococcus):ti,ab,kw OR (Bioflor):ti,ab,kw | 11141 |
|  | #9 | (Akkermansia):ti,ab,kw OR (Lactiplantibacillus):ti,ab,kw OR (Pediococcus):ti,ab,kw OR (Lacticaseibacillus casei):ti,ab,kw OR (Streptococcus):ti,ab,kw | 6589 |
|  | #10 | (Leuconostoc):ti,ab,kw OR (Clostridium butyricum):ti,ab,kw OR (Microbiotas):ti,ab,kw OR (Microbiota):ti,ab,kw OR (Microbiome):ti,ab,kw | 12849 |
|  | #11 | (Microbiomes):ti,ab,kw OR (Mycobiomes):ti,ab,kw OR (Mycobiome):ti,ab,kw | 6600 |
|  | #12 | #6 OR #7 OR #8 OR #9 OR #10 OR #11 | 32637 |
|  | #13 | #4 AND #5 AND #12 | 11 |
| Embase | #4 | #1 AND #2 AND #3 | 484 |
|  | #3 | 'intestines'/exp OR intestines OR intestine OR intestinal OR gut | 1257349 |
|  | #2 | 'reperfusion injury'/exp OR 'reperfusion injury' OR (('reperfusion'/exp OR reperfusion) AND ('injury'/exp OR injury)) OR (reperfusion AND injuries) OR (injury, AND 'ischemia reperfusion') OR (injury, AND ischemia AND reperfusion) OR ('ischemia reperfusion' AND injuries) OR (injury, AND reperfusion) OR ('ischemia reperfusion' AND injury) OR (ischemia AND reperfusion AND injury) OR (reperfusion AND damage) OR (damage, AND reperfusion) OR (reperfusion AND damages) OR (ischemia AND reperfusion) OR 'ischemia reperfusion' | 140336 |
|  | #1 | 'probiotics'/exp OR probiotics OR probiotic OR prebiotics OR synbiotics OR symbiotic OR lactobacillus OR lactobacillaceae OR lactococcus OR bacillus OR clostridium OR saccharomyces OR bifidobacterium OR enterococcus OR bioflor OR akkermansia OR lactiplantibacillus OR pediococcus OR (lacticaseibacillus AND casei) OR streptococcus OR leuconostoc OR (clostridium AND butyricum) OR microbiotas OR microbiota OR microbiome OR microbiomes OR mycobiomes OR mycobiome | 886801 |
| Scopus |  | ( ( TITLE-ABS-KEY ( probiotics ) OR TITLE-ABS-KEY ( probiotic ) OR TITLE-ABS-KEY ( prebiotics ) OR TITLE-ABS-KEY ( synbiotics ) OR TITLE-ABS-KEY ( symbiotic ) OR TITLE-ABS-KEY ( lactobacillus ) OR TITLE-ABS-KEY ( lactobacillaceae ) OR TITLE-ABS-KEY ( lactococcus ) OR TITLE-ABS-KEY ( bacillus ) OR TITLE-ABS-KEY ( clostridium ) OR TITLE-ABS-KEY ( saccharomyces ) OR TITLE-ABS-KEY ( streptococcus ) OR TITLE-ABS-KEY ( bifidobacterium ) OR TITLE-ABS-KEY ( enterococcus ) OR TITLE-ABS-KEY ( bioflor ) OR TITLE-ABS-KEY ( akkermansia ) OR TITLE-ABS-KEY ( lactiplantibacillus ) OR TITLE-ABS-KEY ( pediococcus ) OR TITLE-ABS-KEY ( lacticaseibacillus AND casei ) OR TITLE-ABS-KEY ( streptococcus ) OR TITLE-ABS-KEY ( leuconostoc ) OR TITLE-ABS-KEY ( clostridium AND butyricum ) OR TITLE-ABS-KEY ( microbiotas ) OR TITLE-ABS-KEY ( microbiota ) OR TITLE-ABS-KEY ( microbiome ) OR TITLE-ABS-KEY ( microbiomes ) OR TITLE-ABS-KEY ( mycobiomes ) OR TITLE-ABS-KEY ( mycobiome ) ) ) AND ( ( TITLE-ABS-KEY ( reperfusion AND injury ) OR TITLE-ABS-KEY ( reperfusion AND injuries ) OR TITLE-ABS-KEY ( injury, AND ischemia-reperfusion ) OR TITLE-ABS-KEY ( injury, AND ischemia AND reperfusion ) OR TITLE-ABS-KEY ( ischemia-reperfusion AND injuries ) OR TITLE-ABS-KEY ( injury, AND reperfusion ) OR TITLE-ABS-KEY ( ischemia-reperfusion AND injury ) OR TITLE-ABS-KEY ( ischemia AND reperfusion AND injury ) OR TITLE-ABS-KEY ( reperfusion AND damage ) OR TITLE-ABS-KEY ( damage, AND reperfusion ) OR TITLE-ABS-KEY ( reperfusion AND damages ) OR TITLE-ABS-KEY ( ischemia AND reperfusion ) OR TITLE-ABS-KEY ( ischemia-reperfusion ) ) ) AND ( ( TITLE-ABS-KEY ( intestines ) OR TITLE-ABS-KEY ( intestine ) OR TITLE-ABS-KEY ( intestinal ) OR TITLE-ABS-KEY ( gut ) ) ) | 415 |

Supplementary Table 2. Factors detected in probiotic-protected intestinal IRI for all included articles

| Name |  | Detected factors |
| --- | --- | --- |
| Jakesevic 2011 |  | (Intestinal tissue) MDA  Lactobacillus |
| Wang 2013 |  | Enterobacteria, Enterobacteria, Bacteroides, bifidobacterial, Lactobacillus  SCFA, Acetate, Propionate, Butyrate, Valerate  (serums) endotoxin, TNF-α, IL-6  Chiu's score  claudin-1, occludin, ZO-1 |
| Duranti 2018 |  | (Intestinal tissue) MPO, MDA  Chiu's score |
| Hu 2022 |  | Lactobacillus murinus, FITC-dextran  (Intestinal tissue) Chiu's score, occludin mRNA, ZO-1 mRNA, occludin, ZO-1 |
| Wang 2011 |  | (Intestinal tissue) Chiu's score  (serums) TNF-a, IL-10, IL-6  Enterococcus, Lactobacillus, Bifidobacteria, Bacteroides |
| Håkansson 2006 |  | Enterobacteriaceae,  (Intestinal tissue) MDA |
| Salim 2013 |  | (plasma) IL-1β, TNF-α, IL-6, IL-10  (Intestinal tissue) MPO, IL-1β, |
| Gao 2024 |  | (Intestinal tissue) lipopolysaccharide(LPS), TNF-a, IL-6, IL-1β, IL-10，occludin, ZO-1, FITC-dextran |
| Takayama  2024 |  | Prevotella, Bacteroides, Lactobacillales, Clostridium subcluster XIVa, Clostridium cluster XI, Clostridium cluster XVIII, IgA |
| Tang 2023 |  | (serums)IL-1, IL-1β, IL-6, IL-4, IL-10, TNF-a, CRP, D-LAC, DAO, GSH, MDA, SOD  (Intestinal tissue) Occludin, ZO-1, Claudin mRNA, MUC2 mRNA, ROS, Nrf2, keap1, HO-1, Gpx4, bcl-2, bax |
| Tang 2024 |  | (serums)IL-1, IL-1β, IL-6, IL-4, IL-10, TNF-a, CRP, D-LAC, DAO, IKB-α mRNA, p65 mRNA, IL-6 mRNA, Tnf-α mRNA, MDA, GSH, SOD (Intestinal tissue)Occludin, ZO-1, Claudin mRNA, MUC2 mRNA, p-p65, p65, p-p65/p65, ROS, Nrf2, keap1, HO-1, Gpx4m bcl-2, bax, caspase-3, Nrf2 mRNA, HO-1 mRNA, iNOS mRNA |
| Chen 2024 |  | (serums) FITC-dextran, I-FABP  (Intestinal tissue) Chiu's score, Occludin, ZO-1, KC, TGF-β1, IL-18, IL-23, MDC, IL-10, IL-12p70, IL-6, TNF-α, G-CSF, TARC, IL-12p40, IL-1β |
